# Worsening premature death burden gap from systemic sclerosis in men and black persons: A US nationwide population-based study

**DOI:** 10.1177/23971983221140538

**Published:** 2022-12-08

**Authors:** Ram Raj Singh, Devanshu R Singh, Eric Y Yen

**Affiliations:** 1 Autoimmunity and Tolerance Laboratory, Division of Rheumatology, Department of Medicine, University of California at Los Angeles (UCLA), David Geffen School of Medicine, Los Angeles, CA, USA; 2 Molecular Toxicology Interdepartmental Program, University of California at Los Angeles (UCLA), Los Angeles, CA, USA; 3 Jonsson Comprehensive Cancer Center, University of California at Los Angeles (UCLA), Los Angeles, CA, USA; 4 Department of Pathology and Laboratory Medicine, University of California at Los Angeles (UCLA), Los Angeles, CA, USA; 5 Johns Hopkins Whiting School of Engineering, Baltimore, MD, USA

**Keywords:** Race, sex, systemic sclerosis, large database, mortality

## Abstract

**Objective:**

Male sex and black race incur poor prognosis in systemic sclerosis (SSc). There is no nationwide population-based assessment of premature SSc death burden by sex and race.

**Methods:**

This is a population-based study comprising all recorded SSc deaths across the United States. We constructed histograms depicting the number of SSc deaths for each age by sex and race, and calculated the cumulative percent death at each age and the median age of death. We determined the odds ratios for the risk of premature death from SSc by sex and race. We then calculated the percent of total SSc deaths for different age groups by sex and race from 1970 to 2015. We performed chi-square test with Yates’s correction and quantified the odds ratio (OR) with 95% confidence interval (CI).

**Results:**

The median age of SSc death was 63 years in males versus 68 years in females, and 57 years in blacks versus 70 years in whites. The odds for SSc death before 65 years age was 1.8 (95% CI, 1.6–2.0) for males compared with females and 5.1 (95% CI, 4.4–6.0) for blacks compared with whites. The higher odds for premature death in males than in females was similar for both races. Differences in the proportions of premature deaths from 1970 to 2015 increased between males and females (−5% to 17%) and between blacks and whites (14% to 36%).

**Conclusion:**

Males and black persons die of SSc at younger ages. The worsening premature death burden gap between the two sexes and races over the last five decades is troublesome.

## Introduction

Systemic sclerosis (SSc) is more common in women than men,^1^ which is reflected in the higher age-standardized mortality rate for SSc in women than men.^2^ However, standardized mortality ratios for SSc were found to be similar between men and women^3,4^ or higher in men than women.^5^ Men with SSc also seem to have more years of potential life lost^5^ and a lower 10-year survival than women with SSc.^6^ Thus, men with SSc may die at younger ages. SSc mortality also varies by race, with higher mortality rates in black persons than white persons.^2,7,8^ Black persons also have a higher SSc incidence, more severe diffuse disease, poor prognostic factors, and younger age at disease onset compared with non-black patients,^9,10^ which may cause death at younger ages in black persons. There are no large population-based studies across the United States on premature death from SSc by sex and race.

Most previous studies of SSc outcome by sex and race were based primarily on deaths in patient cohorts at referral centers or in small populations,^5,9–12^ which does not capture the true burden and changes in SSc outcome over time in the general population. These limitations may have contributed to inconsistent findings across previous studies. We used the Centers for Disease Control and Prevention’s (CDC) national mortality database that encompasses more than 99% deaths of US residents in all 50 states and the District of Columbia to determine (a) the median age of SSc death by sex and race, (b) odds ratios for the risk of SSc deaths by sex and race in different age groups, and (c) trends in the proportions of total SSc deaths by sex and race at different age groups over 5 decades.

## Methods

We used the CDC WONDER (Wide-ranging Online Data for Epidemiologic Research) web application to gather data on SSc deaths, as described previously.^2^ Deaths were attributed to SSc if an International Classification of Diseases (ICD) code for SSc was listed as the underlying cause of death on the death certificates (ICD-8, 734.0; ICD-9, 710.1; and ICD-10, M34). Age and race were ascertained using standard methods.^13^ Race has been classified as white, black or African American, and “other.” Information on Hispanic ethnicity and on Asian or Pacific Islander, or American Indian or Alaska Native racial categories is not available in the CDC WONDER before 1999.

We constructed histograms that depict the absolute number of SSc deaths for each age separately by sex and race for years 2011 through 2015. We then assessed the cumulative percent death at each age and determined the median age at death for each demographic group. We also computed the number of SSc deaths during 2011–2015 by sex and race in 0–64 and ⩾65 years age groups.

Next, we obtained the annual numbers of SSc deaths in different age groups by sex and race every 10 years since 1970 and at the end of the study, that is, 2015. We used these numbers to calculate the percent of total SSc deaths by sex and race for different age groups at the selected years of death.

To determine whether the differences in the proportions of SSc deaths between men and women and between black and white persons in different age groups is more than expected by chance, we performed chi-square statistics with Yates’s correction (GraphPad Prism 6.07) and quantified the odds ratio with its 95% confidence interval (CI).

## Results

### Age distribution of SSc deaths according to sex and race

**Figure 1.**
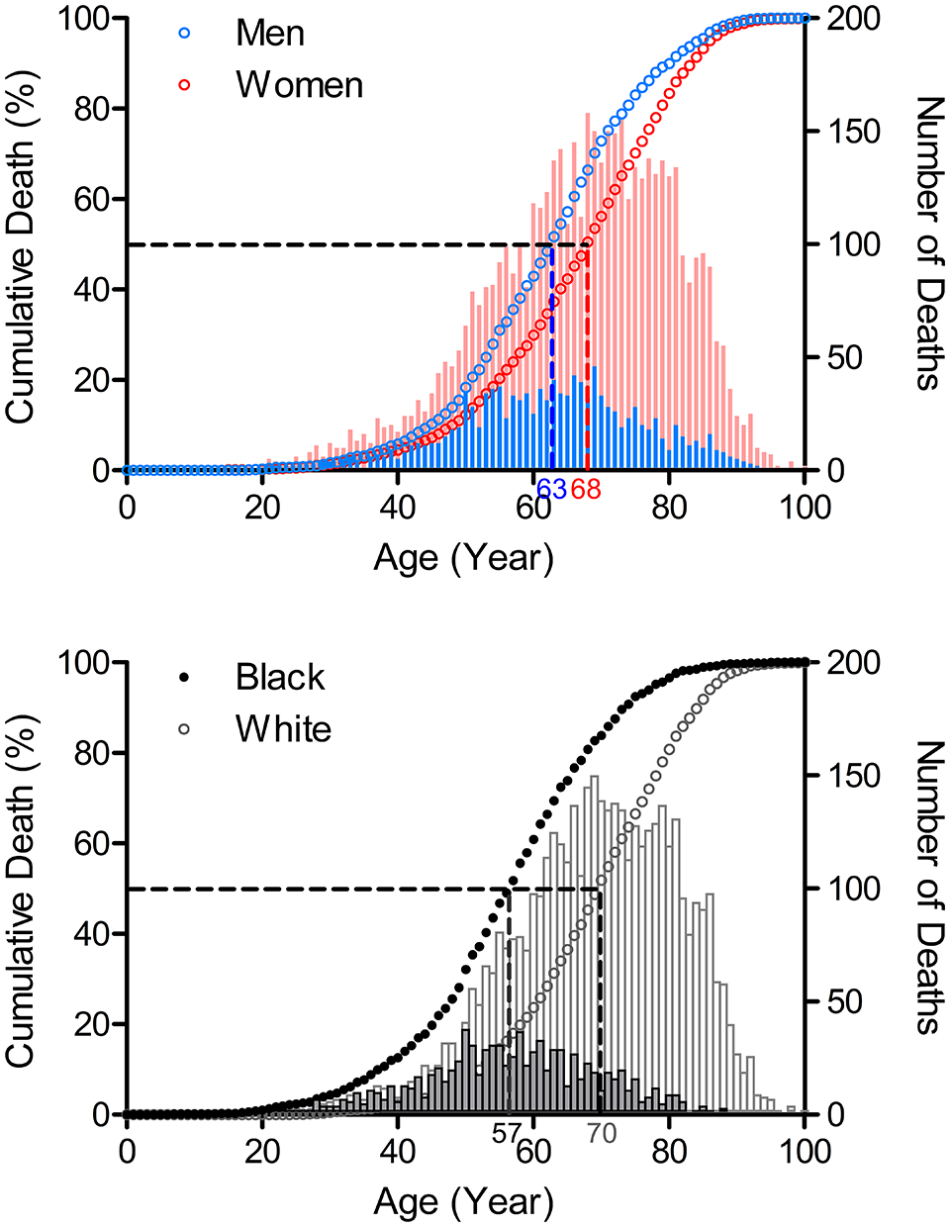
Age distribution of SSc deaths by sex and race, 2011–2015. Vertical bars represent the total number of SSc deaths (Y axis, right) at each year of age (X axis). Symbols represent the percent of cumulative deaths (Y axis, left) at each age. Vertical dashed lines indicate the age by which 50% of the total SSc deaths occurred: men 63 years and women 68 years; black persons 57 years, white persons 70 years.

From 2011 through 2015, SSc was recorded as the cause of death in 5061 females and 1222 males in the United States. Among those deceased of SSc, 4426 were white and 956 black persons. The median age of SSc death was 63 years in males versus 68 years in females and 57 years for black versus 70 years for white persons (Figure 1).

Higher proportions of males than females (54.5% vs 40.2%) and of black than white persons (72.5% vs 34.0%) died of SSc before 65 years of age. The odds of SSc death before 65 years of age was significantly higher in males than in females (odds ratio, 1.8; 95% CI, 1.6–2.0, *p* < 0.001) and in black persons than in white persons (odds ratio, 5.1; 95% CI, 4.4–6.0, *p* < 0.0001) (Table 1).

**Table 1.** Distribution of SSc deaths by age groups according to sex and race, 2011–2015.

Characteristic	<65^a^		⩾65^a^		Odds ratio (95% CI)^b^	*p*
	No.	%	No.	%		
Sex						
Females	2034	(40.2)	3027	(59.8)		
Males	666	(54.5)	556	(45.5)	1.8 (1.6–2.0)	<0.0001
Race						
White	1506	(34.0)	2920	(66.0)		
Black	693	(72.5)	263	(27.5)	5.1 (4.4–6.0)	<0.0001

Abbreviations: CI, confidence interval; SSc, systemic sclerosis.

a Values are numbers of SSc deaths from 2011 through 2015 from all 50 states and the District of Columbia (percentages of death of the respective demographic group) for non-Hispanic white and black persons.

b Chi-square statistics with Yates’s correction to investigate whether the differences between earlier deaths (<65 years of age) in males (vs females) and blacks (vs whites) is more than expected by chance.

Next, we calculated the odds ratios for early SSc deaths (<65, <55, <45, and <35 years) for males compared with females in black and white persons (Table 2). The significantly higher odds of death before 55 and 45 years in males than in females were similar for white and black persons: 1.9 and 1.6 (*p* < 0.0001 and 0.01) for whites and 1.5 and 1.7 (*p* < 0.01) for blacks. The odds of SSc death before 65 years of age in males than in females was statistically significant in white (odds ratio, 2.0; 95% CI, 1.7–2.3, *p* < 0.0001), but not in black persons (odds ratio, 1.4; 95% CI, 0.96–2.0, *p* = 0.1).

**Table 2.** Distribution of SSc deaths by age groups according to sex in black and white persons, 2011–2015.

Race	Sex	No.^a^	%^a^	No.	%	Odds ratio (95% CI)^b^	*p*
		**<65**		**⩾65**			
White	Females	1109	(28.9)	2723	(71.1)		
	Males	397	(44.7)	492	(55.3)	2.0 (1.7–2.3)	<0.0001
Black	Females	525	(70.7)	218	(29.3)		
	Males	168	(76.7)	51	(23.3)	1.4 (0.96–2.0)	0.1
		**<55**		**⩾55**			
White	Females	402	(11.2)	3179	(88.8)		
	Males	165	(19.5)	680	(80.5)	1.9 (1.6–2.3)	<0.0001
Black	Females	304	(41.2)	434	(58.8)		
	Males	112	(51.4)	106	(48.6)	1.5 (1.1–2.0)	<0.01
		**<45**		**⩾45**			
White	Females	118	(3.3)	3463	(96.7)		
	Males	44	(5.2)	801	(94.8)	1.6 (1.1–2.3)	0.01
Black	Females	119	(16.1)	619	(83.9)		
	Males	52	(24.6)	166	(75.4)	1.7 (1.2–2.4)	< 0.01
		**<35**		**⩾35**			
White	Females	38	(1.1)	3543	(98.9)		
	Males	13	(1.5)	832	(98.5)	1.5 (0.8–2.8)	0.3
Black	Females	50	(6.8)	688	(93.2)		
	Males	19	(8.7)	199	(91.3)	1.3 (0.8–2.3)	0.4

a Values are numbers of SSc deaths from 2011 through 2015 from all 50 states and the District of Columbia (percentages of death of the respective demographic group) for non-Hispanic white and black persons.

b Chi-square statistics with Yates’s correction to investigate whether the differences between earlier deaths (<55 or <45 years of age) in males (vs females) for white and black persons is more than expected by chance.

## Differences in percent deaths by sex and race for different age groups over time

**Figure 2.**
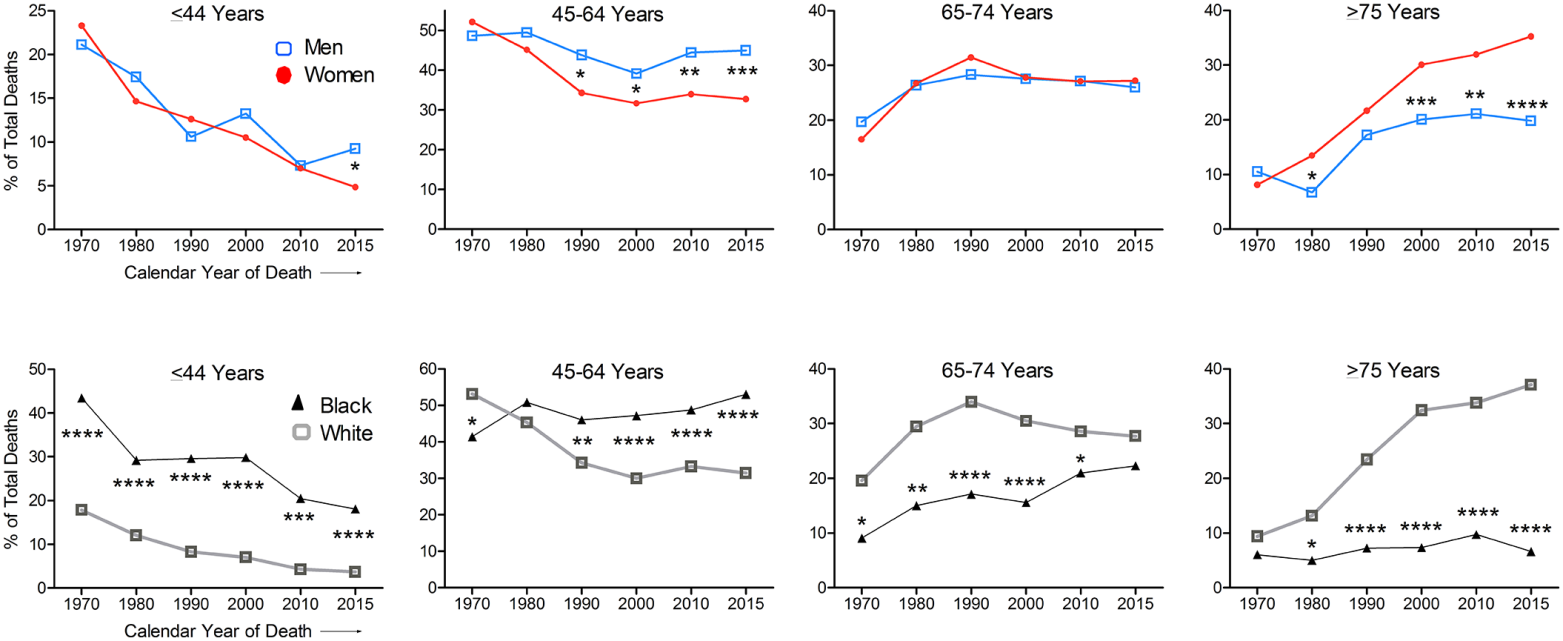
Annual percent SSc deaths in different age groups by sex and race. The percent of annual SSc deaths in the four age groups were calculated separately for each sex and race. Data are displayed for selected years of death (every 10 years since 1970 and in 2015). The annual numbers of SSc deaths in different age groups by sex and race at these years are provided in Supplemental Table S1. *p < 0.05, **p < 0.01, ***p < 0.001, and ****p < 0.0001, chi-square test with Yates correction. Information on Hispanic ethnicity and on Asian or Pacific Islander, or American Indian or Alaska Native racial categories is not available before 1999 and is therefore not shown.

To assess whether the above differences in the proportion of SSc deaths by sex and race have changed over time, we obtained the annual numbers of SSc deaths in different age groups by sex and race (Supplemental Table S1). The percent of annual SSc deaths in the four age groups were calculated separately for each sex and race for selected years of death (every 10 years since 1970 and in 2015 (the latest available data point at the time of analysis)) (Figure 2). The proportions of male and female SSc deaths were similar in all age groups in 1970, but significantly higher proportions of male SSc deaths relative to female SSc deaths were noted in the younger age groups at later timepoints (1990 and later in 45–64, and 2015 in ⩽44 age groups).

The proportions of all premature (0–64 years age) SSc deaths was 70% in males versus 75% in females in 1970, a male-to-female gap of −5% (*p* = NS). This trend reversed in 1980 and has worsened since then. In 2015, 54% SSc deaths in males versus 37% in females occurred before 65 years age, a gap of +17% (*p* < 0.001). The male-to-female gap in the proportions of all SSc deaths before 75 years age was −3% in 1970 to +15% in 2015.

Compared with white persons, black persons had higher percentages of total SSc deaths in ⩽44 years age group throughout the study period (*p* < 0.001). In the 45–64 years age group, SSc deaths were lower in 1970 (*p* < 0.05), not different in 1980, but significantly higher and increasing from 1990 onward in black than white persons (*p* < 0.01 to < 0.0001) (Figure 2).

The proportions of all premature (0–64 years age) SSc deaths were 85% in blacks and 71% in whites, a difference of 14% in 1970. This racial gap increased by 7%–10% every 10 years to 40% (77% in blacks and 37% in white) in 2000 and plateaued after that. In 2015, 71% of SSc deaths in blacks as compared with 35% in whites occurred before 65 years age, a gap of 36%.

The proportions of SSc deaths before 75 years age have decreased from 91% in 1970 to 63% in 2015 in white persons; however, it has remained 90%–95% in black persons over a 45-year period. The racial gap in SSc deaths <75 years age has increased from 3% in 1970 (*p* = NS) to 30% in 2015 (*p* < 0.0001).

## Discussion

Analyses of all deaths recorded across the United States from 2011 through 2015 revealed that males and black persons died of SSc at younger ages than did females and white persons with the median-age-at-death difference of 5 and 13 years, respectively. The odds of dying from SSc before 65 years of age was 1.8 times higher for males than females and 5.1 times higher for black than white persons. The higher odds of early SSc death in males than in females was similar for black and white persons. Furthermore, differences in the proportions of premature SSc deaths between males and females and between black and white persons have worsened over the last five decades.

SSc is ~4.6 times more prevalent in women than men in the United States (389.8 vs 84.1 per million),^1^ while age-standardized mortality rate is only 3.5-fold higher in women than men (4.9 (4.6–5.2) vs 1.4 (1.2–1.5) per million).^2^ Thus, after controlling for prevalence men may experience a higher SSc mortality than women. Indeed, survival at 10 years from the onset of SSc symptoms was lower (75.3%) for men than women (92.9%),^6^ and men experienced 3.6 more years of potential life lost than women with SSc.^5^ Despite a lower mean age at diagnosis in women than men in most studies,^14,15^ we report that men with SSc died at younger ages than women with SSc across the United States. This could be due to more frequent diffuse disease with rapid progression, more frequent and severe lung fibrosis, and more severe peripheral vascular disease in men than in women.^15,16^ In addition, anticentromere antibodies that are associated with better outcomes in SSc are more frequent in women than men.^2,17^

We further report that higher proportions of premature SSc deaths occurred in men than women in 2015 than in previous decades: The male-to-female gap changed from −5% (1970) to +17% (2015) in <65 years age, and −3% to +15% in <75 years age. Studies are needed to determine whether men with SSc have a more diffuse, rapidly progressive SSc with more lung fibrosis and vascular disease that is less responsive to treatment in the 2000s than in the late-1900s.

SSc is more prevalent in black than in white persons (315.1 (282–352) vs 224.7 (197–256)),^1,14^ so it is not surprising that mortality rates are higher in black persons than white persons.^2,7,8,18^ From 1999 to 2002, SSc death rates peaked a decade earlier in the black population when compared with those in the white population (65–74 vs 75–84 years of age).^18^ In resonance with this, we found that black persons have a 13 years lower median age of SSc death compared with white persons, and a 5.1 times the odds of dying from SSc before 65 years of age. A younger age at disease onset could partly explain the younger age at death from SSc in blacks. For example, the average age was 2 years younger at Raynaud phenomenon onset, and 5 years younger for the first non-Raynaud phenomenon symptom for black than for white patients in a large, 1990–2009, SSc cohort.^19^ Similarly, a 1989–1991 study from Detroit area found the average age at diagnosis 7.1 years younger for blacks than for whites.^1^ Black patients also have a more severe diffuse disease, and lower anticentromere, higher anti-topoisomerase positivity, and other poor prognostic factors compared with non-black patients.^1,9,10,19,20^ Racial differences in the expression of pro-fibrotic and anti-fibrotic factors have also been implicated in the pathogenesis of racial disparity in SSc.^20^ However, in well-characterized and well-cared-for cohorts, Scleroderma Lung Studies I and II, time to death and survival were not different between African American and non-African American participants,^21^ which offers hope that racial disparity in SSc outcome may be amenable to correction, at least in part, by improving access to care by SSc specialists. This study may also suggest that individual, biologic, or genetic factors were less likely to have contributed to racial differences that we observed at the population level.

Disconcertingly, the gap in the proportions of premature SSc deaths between black and white persons have widened over the last five decades: 14% in 1970 to 36% in 2015 (<65 years), and 3% in 1970 to 30% in 2015 (<75 years age). The reasons underlying this profound disparity are unclear. Advances in the early recognition and treatment of SSc and its complications, such as prostanoids, endothelin receptor antagonists, and phosphodiesterase-5 inhibitors to treat pulmonary hypertension, early screening for alveolitis, and selected use of immunosuppressive drugs in SSc lung disease have improved survival.^2^ These advances may have contributed to reduced proportions of SSc deaths <65 years of age from 71% in 1970 to 35% in 2015 in white persons. The worsening racial gap in the proportions of SSc deaths at younger ages over the past 5 decades implies that the current advances in SSc may not be reaching black SSc patients due to non-genetic factors, such as socioeconomic inequality and/or differential healthcare. To assess SSc mortality in the context of higher all-cause mortality in blacks than in whites, we recently computed the ratio of SSc age-standardized mortality rate (ASMR) to non-SSc-ASMR. We found this ratio to be less profoundly different between blacks and whites than the racial gap in SSc-ASMR,^2^ again suggesting possible contributions of non-SSc determinants, such as socioeconomic status and access to care, in imparting higher SSc mortality in black persons.

The use of national mortality database provides an opportunity to assess disease-specific parameters in an unbiased manner using a large sample size, but it has limitations too. Our data likely underestimate the true disease burden in black persons, since under-ascertainment and under-recording of deaths in less-well educated ethnic minorities and uninsured patients are known to occur in autoimmune diseases, as discussed previously.^2,22^ The increased premature SSc deaths in black persons are unlikely to be artifacts from misclassification (i.e. recording SSc as the cause of death on death certificates for decedents that did not have SSc), because greater underreporting of SSc as the cause of death in underprivileged groups would lead to greater underestimation of SSc deaths in this subpopulation. However, we cannot exclude a possibility that changes in physicians’ practice of recognizing SSc and recording it on the death certificates over decades could have partly contributed to changing trends in SSc mortality by sex and race. For example, given difficulty in recognizing Raynaud’s phenomenon in persons with darker skin, musculoskeletal symptoms, skin pigment changes, and positive ANA in some patients with SSc might have been misclassified as lupus in the 1970s–1980s. Improving detection of scleroderma disease over time,^23^ identification of SSc-associated autoantibodies,^24^ and the introduction of classification criteria for early SSc in 2001^25^ might have led to increasing recognition of SSc in black persons at younger ages.

In conclusion, men and black persons with SSc die at younger ages. Despite an overall improvement in SSc mortality in recent years, sex and racial disparities in premature SSc deaths have worsened over the last five decades. Studies are urgently needed to examine mechanisms underlying the disconcerting disparities and to develop research and healthcare programs to reduce it.

## Supplemental Material

sj-pdf-1-jso-10.1177_23971983221140538 – Supplemental material for Worsening premature death burden gap from systemic sclerosis in men and black persons: A US nationwide population-based study Click here for additional data file. Supplemental material, sj-pdf-1-jso-10.1177_23971983221140538 for Worsening premature death burden gap from systemic sclerosis in men and black persons: A US nationwide population-based study by Ram Raj Singh, Devanshu R Singh and Eric Y Yen in Journal of Scleroderma and Related Disorders
