# Supplementary material for: Worsening premature death burden gap from systemic sclerosis in men and black persons: A US nationwide population-based study
Source: J Scleroderma Relat Disord. 2022 Dec 8;8(1):20–6. doi: 10.1177/23971983221140538 (PMC9896199; doi:10.1177/23971983221140538)
Supplement: sj-pdf-1-jso-10.1177_23971983221140538 – Supplemental material for Worsening premature death burden gap from systemic sclerosis in men and black persons: A US nationwide population-based study [file sj-pdf-1-jso-10.1177_23971983221140538.pdf]

Supplemental Materials

Worsening Premature Death Burden Gap from Systemic Sclerosis in Men and Black Persons

A United States Nationwide Population-Based Study

Reproducible Research Statement

The data used in this study were extracted from a database maintained by the Centers for Disease Control and Prevention (CDC), National Center for Health Statistics. The CDC WONDER Online Database contains information compiled from data provided by the 57 vital statistics jurisdictions through the Vital Statistics Cooperative Program.

Supplemental Table S1. Annual SSc Deaths in Different Age Strata by Sex and Race*

Calendar Year of death	Total		≤44 years		45-64 years		65-74 years		≥75 years	
	Men	Women	Men	Women	Men	Women	Men	Women	Men	Women
1970	142	382	30	89	69	199	28	63	15	31
1980	178	512	31	75	88	231	47	137	12	69
1990	226	706	24	89	99	242	64	222	39	153
2000	294	1037	39	109	115	328	81	288	59	312
2010	232	1014	17	71	103	344	63	275	49	324
2015	227	970	21	47	102	317	59	264	45	342
	**Black**	**White**	**Black**	**White**	**Black**	**White**	**Black**	**White**	**Black**	**White**
1970	99	414	43	74	41	220	9	81	6	39
1980	120	563	35	68	61	255	18	166	6	74
1990	152	759	45	63	70	260	26	258	11	178
2000	231	1063	69	75	109	319	36	324	17	345
2010	205	997	42	43	100	332	43	285	20	337
2015	166	989	30	37	88	311	37	274	11	367

* Number of annual SSc deaths, total and by age strata, separately for men and women and for black and white persons in selected years (every 10 years and in 2015).

Information on Asian or Pacific Islander, or American Indian or Alaska Native racial categories and on Hispanic ethnicity is not available before 1999 and is therefore not shown.
